# COVID-19 Patient Psychological Pain Factors

**DOI:** 10.3389/fpsyg.2021.649895

**Published:** 2021-05-19

**Authors:** Niu Zhengkai, Shen Yajing

**Affiliations:** Institute of Analytical Psychology, City University of Macao, Macao, China

**Keywords:** COVID-19, SARS-CoV-2, psychological pain, post-traumatic growth, influencing factors

## Abstract

The level of psychological pain in patients with COVID-19 was investigated in this study by hypothesis testing, one-way ANOVA, multi factor ANOVA, and correlation analysis. The psychological pain thermometer and post-traumatic growth assessment scale were used as research tools. Many factors appear to influence the psychological state of COVID-19 patients including practical problems, communication problems, emotional problems, physical problems, and psychiatric/relative concerns. The severity of the disease, the surrounding environment, family health problems, life perceptions, interpersonal relationships, personal strength, mental changes, new possibilities, and the total post-traumatic growth score are also affected. There is a significant negative correlation between psychological pain and post-traumatic growth. There are significant differences in the degree of psychological pain across the demographic data. Practical problems, communication problems, emotional problems, physical problems, and spiritual/religious concerns show significant effects on the degree of psychological pain.

## Introduction

On January 31, 2020, the World Health Organization (WHO) designated COVID-19 as a global public health emergency (Sohrabi et al., 2020). The virus that caused the emergence of COVID-19 is SARS-CoV-2. SARS-CoV-2 virus particles are mostly round, with capsule, and there are coronal arranged fibrils around them, which are distributed in the cytoplasm. The diameter of SARS-CoV-2 virus is between 80 and 120 nm (Yezhen et al., 2020). According to a recent study in Chinese Journal of epidemiology, it has been found that SARS-CoV-2, like the closely related SARS-CoV, uses a receptor called ACE2 to enter human cells. This entry also requires the assistance of an enzyme called TMPRSS2 (Chinese Journal of Epidemiology, 2020). The infected cell in our body can also send a help signal that it has been infected. It's getting our immune cells here fast. Many of them can work together, such as B cells, plasma cells and dendritic cells. If the infected cells signal help, T cells and natural killer cells respond and kill the infected cells. Our immune system attacks cells infected by the virus, which may lead to many symptoms of colds, influenza and fatal diseases (Giarratana et al., 2021). That's what makes us sick. Generally, patients with COVID-19 are accompanied by “Psychological Pain” for psychological reasons (Li, 2010; Zhanjiang, 2020). Public health emergencies are known to have a massive impact on the psychological states of human beings. Individuals must wear suitable protective clothing (e.g., masks), for example, which has certain psychological effects.To date, the COVID-19 pandemic has seriously affected the physical and mental health of human beings across the globe.

The WHO designated the COVID-19 epidemic as an international public health emergency, as mentioned above. COVID-19 patients suffer from physical pain and bear enormous psychological pressure such as stress reactions (Sohrabi et al., 2020). The disease itself is highly infectious, can be transmitted by asymptomatic carriers, has a long incubation period, and lacks safe, reliable, and effective treatments or preventative vaccines (Lam et al., 2020). An unknown number of COVID-19 cases continues to rise, which creates fear and distress among the general public, COVID-19 patients, and their families and friends.

COVID-19 management is still in its infancy and there has been no official report on psychological pain in COVID-19 patients. In this study, psychological pain levels were assessed on a psychological pain scale. In recent years, researchers of positive psychology have revealed that after an individual experiences a traumatic event, in addition to negative psychological experiences, they show positive psychological changes such as PTG (Yaqin et al., 2011). And this kind of pain is not only physical, but also psychological, which is called “Psychological pain.” “Psychological pain” refers to emotional experiences of psychological (cognitive, behavioral, emotional), social, and (or) spiritual essential importance (Jollant and Olié, 2017). These experiences may affect treatment compliance, physical symptoms, and treatment effects in infectious disease patients (Jollant and Olié, 2017). Post-traumatic growth (PTG) was first defined by Tedeschi, Park, and Calhoun in 1996 as the positive psychological changes experienced by individuals after struggling with traumatic negative life events and situations make individuals and groups grow up from trauma (Tedeschi and Calhoun, 2009). Tsai et al. (2015) observed close correlations between psychological pain and PTG. Arguably, when an individual experiences major events, they may gain positive changes in their perception of self and surroundings; a major event can help one to “reset” and to reprioritize various aspects of one's life. Scholars generally believe that PTG is a positive psychological factor that allows patients to self-renew, self-regulate, recover, and ultimately improve their mental health (Sylwester, 1995). Psychological pain is common in many symptoms of patients with major diseases (Li et al., 2012). The diagnosis of the disease, the course of the disease itself, and the treatment can all create psychological pain in patients (Li et al., 2012). In 2018, the NCCN asserted that it is necessary to comprehensively screen for psychological pain in patients and provide psychological support through multidisciplinary comprehensive treatment groups (Swarm et al., 2019). Tension, worry, and fear are the most common psychological problems in cancer patients (Jing et al., 2011). Zhaoli et al. (2015) found that the top five factors causing psychological pain in cancer patients at admission are economic concerns, worry, sadness, nausea, and trouble eating. COVID-19 management is still in its infancy and there has been no official report on psychological pain in COVID-19 patients. In this study, psychological pain levels were assessed on a psychological pain scale. In recent years, researchers of positive psychology have revealed that after an individual experiences a traumatic event, in addition to negative psychological experiences, they show positive psychological changes such as PTG (Yaqin et al., 2011).

PTG is closely related to psychological pain in major disease patients (Turner-Sack et al., 2012). Yongjun (2015) studied the relationship between PTG, resilience, and disease perception using a PTG rating scale, resilience questionnaire, and revised disease perception questionnaire to investigate 148 patients after neurosurgery. Serfaty et al. (2012) used Pearson correlation analysis to find that the psychological pain of patients with prostate cancer was moderate while the level of PTG was low, showing a negative correlation. This is attributed to the fact that prostate cancer patients are in the middle and late stages when they are diagnosed, are affected by the stress of the disease, and experience side effects of treatment such as androgen reduction, self-image changes, and urethral symptoms that may cause psychological trauma. These studies do not clearly explain the relationship between psychological pain and PTG. Psychological support for patients with advanced lung cancer, for example, has been shown to provide relief of psychological pain, enhancement of PTG, and improved quality of life (Jian et al., 2015). In investigating the psychological pain of COVID-19 patients, the present study was conducted to develop systematic intervention measures to stimulate PTG and reduce psychological pain.

Therefore, this study wants to discuss the main factors of psychological pain in COVID-19 patients, different demographic data is related to the degree of psychological pain, and whether psychological pain is related to PTG. According to the research purpose, we can make the following three hypotheses:

Hypothesis 1: There is a significant correlation between psychological pain and PTG.

Hypothesis 2: There are significant differences in demographic data and the degree of psychological pain.

Hypothesis 3: Practical problems, communication problems, emotional problems, physical problems, and spiritual/religious problems have a significant impact on the degree of psychological pain.

## Research Methodology

The purpose of this chapter is to provide readers with an understanding of the methods employed and related research methods used in our research. In this chapter, we explain the philosophy, methods and Strategies of the research, and why the methodology is adopted, as well as the limitation and limitation of the work related to data collection. Therefore, access to valid data and information is critical. It is particularly important to select appropriate research methods and effectively carry out research to better achieve the research objectives.

COVID-19 patients were selected as subjects in this study. The psychological pain thermometer (DT) (Yining et al., 2010) and post-traumatic growth assessment scale (PTGI) (Yaqin et al., 2011) were used as research tools. A questionnaire was used to gather personal data. A hypothesis test, one-way ANOVA, multi factor ANOVA, correlation analysis, and further investigation were used to determine the degree of psychological pain the subjects experienced and the main influencing factors between psychological pain and PTG.

Four hundred and ninety-six cases of COVID-19 patients were collected from April 2020 to May 2020 as the research object of this study. All COVID-19 patients are treated in local hospitals and do not exist in other places except hospitals. The distribution, isolation and treatment of all patients are provided by the local network. The study has obtained the qualifications to agree to the investigation from the relevant hospitals and the patients' informed notification of the investigation. Some patients are unwilling to accept the questionnaire, the study excluded them. All patients were diagnosed according to criteria in the “Diagnosis and treatment plan of COVID-19 (Trial Eighth Edition)” (Chinese Journal of Clinical Infectious Diseases, 2020) and reached the standard of cure and discharge after treatment.

### Demographic Characteristics and Other Information of Patients

Table 1 shows the demographic characteristics and other information of patients. There were 279 males and 217 females. Among them, 31 people were 16–25 years of age at the time of their participation, accounting for 6.3% of the total. Forty-two (8.5%) were 26–35 years, 59 (11.9%) were 36–45 years, and 137 (27.6%) were 46–55, 103 (20.8%) were 56–65, 85 (17.1%) were 66–75, and 35 (7.9%) were over 75 years old.

**Table 1:** Demographic characteristics and other information of patients (*N* = 496).

**Item**		**Frequency**	**%**
Gender	Male	279	56.3
	Female	217	43.7
Age	16–25	31	6.3
	26–35	42	8.5
	36–45	59	11.9
	46–55	137	27.6
	56–65	103	20.8
	66–75	85	17.1
	>75	39	7.9
Education	Primary school and below	64	12.9
	Junior middle school	177	35.7
	High school	102	20.6
	University and above	153	30.8
Occupation	Farmer	36	7.3
	Worker	72	14.5
	Public institutions or civil servants	98	19.8
	Staff member	207	41.7
	Unemployed	42	8.5
	Other	41	8.3
Monthly income (CNY/USD)	2,000–3,000 CNY (309.6–464.4 USD)	112	22.6
	3,000–5,000 CNY (464.4–774 USD)	203	40.9
	5,000–7,000 CNY (774–1,083.6 USD)	129	26.0
	>7,000 CNY (>1,083.6 USD)	52	10.5
Marriage	Married	296	59.7
	Unmarried	163	32.9
	Widowed	37	7.5
Isolation time (days)	20–30	32	6.5
	30–40	86	17.3
	40–50	285	57.5
	>50	93	18.8
Disease severity	Mild cases	89	17.9
	Common cases	203	40.9
	Severe cases	162	32.7
	Dangerous cases	42	8.5
Information	Full or partial knowledge	199	40.1
	Ignorant of COVID-19	297	59.9
General health condition before COVID-19	Very good	91	18.3
	Relatively good	207	41.7
	Fair	84	16.9
	Relatively poor	63	12.7
	Very poor	51	10.3
Past medical history	Yes	128	25.8
	No	368	74.2
Past psychiatric history	Yes	7	1.4
	No	489	98.6

The sample contained 64 primary school students or below, accounting for 12.9% of the total. There were 177 junior high school students, accounting for 35.7%, and 102 senior high school students, accounting for 20.6%. There were 153 people at university or above, accounting for 30.8%. There were 36 agricultural workers accounting for 7.3% of the total, 72 workers accounting for 14.5%, 98 employees of public institutions or civil servants accounting for 19.8%, 207 staff members accounting for 41.7%, 42 unemployed persons accounting for 8.5%, and 41 marked “other” accounting for 8.3%.

In terms of the subjects' monthly income (CNY/USD), there were 112 people earning 2000-3000 CNY (309.6-464.4 USD) accounting for 22.6% of the total, 203 earning 3,000–5,000 CNY (464.4-774 USD) accounting for 40.9%, 129 earning 5,000–7,000 CNY (774-1083.6 USD) accounting for 26.0%, and 52 earning over 7,000 CNY (>1,083.6 USD) accounting for 10.5%. Two-hundred and ninety-six were married (59.7%) 163 were unmarried (32.9%), and 37 were widows or widowers, accounting for 7.5% of the total.

### Research Procedure

#### Survey Tools

Basic demographic information for the participants was obtained via questionnaire, mainly including age, gender, marital status, number of children, education level, and income. Psychological pain is an unpleasant emotional experience which interferes with patients' responses to cancer, somatic symptoms, and anti-cancer treatment (Li et al., 2012). The NCCN recommends the psychological pain thermometer (DT) as a screening tool to quickly identify the psychological pain of cancer patients (Ransom et al., 2006). The scale can be completed in about 3 min and is widely used in cancer clinics. Lili et al. (2010) confirmed that the DT is suitable for Chinese cancer patients. They revised the foreign version of the DT to establish a Chinese DT. The degree of psychological pain was evaluated via DT in this study over a scale from 0 to 10, where 0 indicates no pain, 1–3 indicates mild pain, 4–6 indicates moderate pain, 7–9 indicates severe pain, and 10 indicates extreme pain. A higher score relates to a larger degree of psychological pain. The Cronbach index was 77.0, which indicates high validity.

The post-traumatic growth inventory (PTGI) was used to evaluate PTG over five dimensions: life perception, interpersonal relationships, personal strength, mentality changes, and perception of new possibilities (Ji et al., 2011). There are 20 items in total. Each is scored between 0 and 5 points to make a total score between 0 and 100; a higher total score indicates a higher level of PTG (<60 points for low level, 60–79 points for medium level, and ≥80 points for high level) (Ji et al., 2011). The Cronbach's α coefficient of the scale is 0.875 and the validity index is 0.859, indicating high reliability and validity (Tedeschi and Calhoun, 1996).

Ji et al. (2011) introduced PTGI into the psychological evaluation and intervention of accidental trauma patients in China to promote the physical and mental recovery of the injured from an innovative perspective. The PTGI was translated back, culturally adjusted, and semantically analyzed for use in the accidental trauma patients to test its reliability and validity. Factor analysis reveals five dimensions. The scale has good reliability and validity and is suitable for the study of PTG in Chinese populations.

#### Survey Method

The participants obtained permission from the relevant authorities before the study was carried out and all provided informed consent. The questionnaires were distributed in April 1st 2020; the participants completed them on site and returned them immediately. The duration is 2 weeks, until April 15, 2020. All surveys are filled out on the spot using paper and pen. The staff perform formal operations and wear protective clothing to solve the patients' questions that may arise during the investigation. The health staff explained the purpose of the survey to the participants in detail when distributing the questionnaire and provided immediate answers to any questions the participants had in filling them out. A total of 613 questionnaires were distributed, among which 550 were returned and 496 were deemed valid (effective rate of recovery = 90.18%).

#### Quality Control Methods

As mentioned above, the staff explained the questionnaire requirements and confidentiality principles to the participants on site to encourage them to give serious, anonymous, and authentic responses. The questionnaire data was sorted and entered into an Excel worksheet for further analysis by two staff members to cross-check its correctness.

#### Statistical Methods

Two staff members input and analyzed the data by first transforming it into SPSS files using EpiData 3.1 software before processing it in Statistical Product and Service Solutions (SPSS) 19.0. Statistical analysis was performed using SPSS as well (Yuguang et al., 2014). The basic data, psychological DT, and PTG assessment scales were input as calculation frequency, percentage, mean value, and standard deviation values. Pearson correlation was used to determine the correlation between the psychological pain score and PTG score. **Table 4** uses demographic data such as Gender, Age, Education, Occupation, Monthly income (CNY/USD), Marriage, Isolation time (days), Disease severity, Information, General health condition before COVID-19, Past medical history and Past psychiatric history Academic information as relevant factors. Through the process of hypothesis testing, we can judge whether many factors have a significant impact on the dependent variable. In the multivariate analysis of variance, there are many factors that affect the dependent variables, some of which may affect the dependent variables in addition to their own. Using multi-factor analysis of variance and psychological pain to make a comparison, so as to prove whether there are significant differences between them. In **Table 5**, severity of COVID-19 was used as the independent variable. The related factors like practical problems, communication problems, emotional problems, physical problems, and spiritual/religious concerns were analyzed by chi square. This can count the deviation degree between the actual observation value and the theoretical inference value, and the deviation degree between the actual observation value and the theoretical inference value determines the chi square value. This analysis can derive the correlation between these factors and the severity of COVID-19. If *p* < 0.05, the difference was considered statistically significant.

## Results

In Table 1, thirty-two patients (6.5%) were isolated for 20–30 days, 86 (17.3%) for 30–40 days, 385 (57.5%) for 40–50 days, and 93 people for more than 50 days, accounting for 18.8% of the total. There were 89 mild cases (17.9%), 203 common cases (40.9%), 162 severe cases (32.7%), and 42 dangerous cases (8.5%). Almost 200 people (40.1%) fully understood COVID-19 and 297 were unaware of the disease, accounting for 59.9% of the total. Ninety-one subjects (18.3%) were in good general health before their diagnosis, 207 (41.7%) were in relatively good health, 84 (16.9%) were in fair health, 63 (12.7%) were in relatively poor health, and 51 (10.3%) were in very poor health. One-hundred and twenty-eight (25.8%) had previous medical history and 368 (74.2%) had no medical history prior to their COVID-19 diagnosis. Further, seven subjects had a history of mental health treatment, accounting for 1.4% of the sample; 489 (98.6%) had no history of mental illness.

### COVID-19 Patients' Psychological Pain and Post-traumatic Growth

Table 2 lists the psychological pain and PTG scores of the subjects. The higher the score, the more posttraumatic growth. The average scores of psychological pain and PTG were 4.53 ± 2.28 and 53.6 ± 7.69, respectively. In the three dimensions of PTG, the score of interpersonal relationships was the highest (16.87 ± 4.96), followed by life perception (11.31 ± 3.27), personal power (10.19 ± 3.17), and spiritual change (8.02 ± 1.99). The score of new possibilities was the lowest (7.21 ± 1.63).

**Table 2:** COVID-19 patients' psychological pain and PTG ($\bar{\chi }\pm s$ sub).

**Index**	**Dimension**	**Scoring results**
Psychological pain	4.53 ± 2.28	
PTG	Perception of life	11.31 ± 3.27
	Interpersonal relationships	16.87 ± 4.96
	Personal power	10.19 ± 3.17
	Spiritual change	8.02 ± 1.99
	New possibilities	7.21 ± 1.63
	Total score	53.6 ± 7.69

Table 3 shows that the sampled COVID-19 patients' psychological pain was negatively correlated with their total PTG score. There was a significant negative correlation between the total score of PTG and psychological pain. The correlation coefficient is −0.713. As for the five dimensions of PTG, perception of life was negatively correlated with psychological pain, and the correlation coefficient was −0.501. There was a significant negative correlation between psychological pain and interpersonal. There was a significant negative correlation between personal power and psychological pain, and the correlation coefficient was −0.465. There was a significant negative correlation between spiritual changes and psychological pain, and the correlation coefficient was −0.574. There was a significant negative correlation between new possibilities and psychological pain, and the correlation coefficient was −0.496.

**Table 3:** Pearson correlation analysis between COVID-19 patients' psychological pain and PTG.

**Index**		**Psychological pain**
Perception of life	Pearson	−0.501^**^
Interpersonal relationships	Pearson	−0.488^*^
Personal power	Pearson	−0.465^**^
Spiritual changes	Pearson	−0.574^***^
New possibilities	Pearson	−0.496^*^
Total PTG score	Pearson	−0.713^*^

* *p < 0.05*,

** *p < 0.01*,

*** *p < 0.001*.

### Factors Influencing COVID-19 Patients' Psychological Pain

#### Comparison Based on Demographic Characteristics and Pearson Correlations

Table 4 shows a significant difference in the psychological pain score of patients in the demographic variable of gender. *p* < 0.05.

**Table 4:** Comparison analysis of COVID-19 patients' psychological pain.

**Project**		**Psychological pain score**	***t*/F**	***r***
Gender	Male	5.07 ± 3.32	3.994^*^	\
	Female	3.96 ± 2.77		
Age	16–25	2.96 ± 3.65	1.261^*^	0.326^*^
	26–35	3.17 ± 2.41		
	36–45	5.97 ± 3.09		
	46–55	6.87 ± 2.54		
	56–65	5.34 ± 4.31		
	66–75	4.99 ± 4.68		
	>75	4.66 ± 2.84		
Education	Primary school	5.42 ± 2.97	1.214^**^	−0.291^**^
	Junior middle school	4.41 ± 3.01		
	High school	4.05 ± 2.88		
	University and above	3.51 ± 3.31		
Occupation	Farmer	4.07 ± 3.26	0.074	\
	Worker	3.99 ± 3.71		
	Public institutions or civil servants	4.32 ± 2.91		
	Staff member	3.89 ± 2.52		
	Unemployed	3.80 ± 2.95		
	Other	4.21 ± 2.17		
Monthly income (CNY/USD)	2,000–3,000 CNY (309.6–464.4 USD)	4.82 ± 3.65	0.983^**^	−0.433^**^
	3,000–5,000 CNY (464.4–774 USD)	4.12 ± 2.49		
	5,000–7,000 CNY (774–10,83.6 USD)	3.28 ± 2.01		
	>7,000 CNY (>1,083.6 USD)	3.52 ± 2.77		
Marriage	Married	4.27 ± 3.03	1.755	\
	Unmarried	5.48 ± 4.79		
	Widowed	1.86 ± 0.78		
Isolation time (days)	20–30	3.31 ± 2.64	3.143^**^	0.601^**^
	30–40	3.96 ± 2.77		
	40–50	5.09 ± 2.62		
	>50	6.71 ± 3.41		
Disease severity	Mild cases	1.81 ± 2.47	12.735^***^	0.552^**^
	Common cases	4.11 ± 2.82		
	Severe cases	3.57 ± 2.39		
	Dangerous cases	7.73 ± 3.01		
Information	Full or partial knowledge	4.29 ± 3.07	2.327^**^	\
	Ignorant of COVID-19	2.21 ± 1.91		
General health condition before COVID-19	Very good	2.48 ± 1.99	3.553^**^	0.570^**^
	Relatively good	4.29 ± 2.36		
	Fair	3.74 ± 1.65		
	Relatively poor	5.89 ± 3.70		
	Very poor	6.02 ± 3.79		
Past medical history	Yes	4.77 ± 2.15	1.963	\
	No	3.96 ± 2.54		
Past psychiatric history	Yes	6.28 ± 3.82	2.896^**^	\
	No	3.91 ± 1.70		

* *p < 0.05*,

** *p < 0.01*,

*** *p < 0.001*.

There is also a significant difference in psychological pain scores among the three age groups (at *p* < 0.05). The three age groups with the highest scores were 46–55, 36–45, and 56–65 years. Pearson correlation analysis showed a significant positive correlation between age and psychological pain with a coefficient of 0.326.

There was a significant difference in the psychological pain score between two groups (*p* < 0.01) in terms of income. Pearson correlation analysis results show a significant negative correlation between income and psychological pain at a coefficient of −0.433.

There was a significant difference in terms of isolation time as well (*p* < 0.01). The psychological pain score of patients who isolated for more than 50 days was significantly higher than that of other groups. Pearson correlation analysis showed a significant positive correlation between the duration of isolation and psychological pain with a coefficient of 0.60.

There was a significant difference in psychological pain scores in terms of disease severity as well (*p* < 0.001). The psychological pain score of patients in the dangerous case group was significantly higher than that of other groups. Pearson correlation analysis showed a significant positive correlation between disease severity and psychological pain with a correlation coefficient of 0.552.

The patients' educational background and knowledge of COVID-19 also produced significant differences in psychological pain scores (*p* < 0.01). Pearson correlation analysis showed that the degree of education was negatively correlated with the intensity of psychological pain at a coefficient of −0.29.

The patients' general health condition before COVID-19 also produced significant differences in their psychological pain levels (*p* < 0.01). COVID-19 was negatively correlated with the general health condition prior to diagnosis at a coefficient of 0.570.

There was no significant difference in terms of career, marital status, or past medical history among the subjects.

#### Related Factors Potentially Affecting Psychological Pain

The following factors related to psychological pain are all taken from the psychological pain thermometer. The investigation of COVID-19 patients by using the psychological pain thermometer can analyze the factors that affect the patients' psychological pain.

The psychological pain of 496 patients may originate in problems related to the care of children and old adults dependents (18.6%), housing (9.4%), insurance/financial situations (36.7%), transportation (2.1%), work/school (61.5%), and the surrounding environment (51.0%).

Emotional problems include depression (58.2%), fear (55.2%), loneliness (48.5%), nervousness (51.8%), sadness (12.7%), worry (52.8%), loss of interest in daily activities (46.2%), sleep issues (60.9%), and memory loss/inattention (18.3%).

Physical problems include appearance/bodily form (5.0%), bathing/dressing (7.6%), breathing (57.2%), micturition changes (2.6%), physical constraints (7.8%), diarrhea (1.4%), eating issues (6.3%), tiredness (28.6%), edema (1.0%), fever (72.8%), dizzy (8.2%), indigestion (9.1%), pain existing previously to disease onset (5.4%), nausea (2.0%), dry mucous membranes (5.4%) or congestion (60.1%), pain related to the disease (19.2%), sexual dysfunction (2.0%), dry or itchy skin dry (0.8%), numbness of hands/feet (3.4%), and limited physical activity(3.0%). Spiritual/religious concerns also have noteworthy effects (2.4%).

A Chi square test was used to probe whether the patients had significant psychological pain related to these items. The results are shown in Table 4.

Actual problems affecting the subjects included child and old adults care (18.6%), housing (9.4%), insurance/financial (36.7%), transportation (2.1%), work/school (61.5%), and the surrounding environment (51.0%).

Communication with children/old adults (10.4%), partners (19.8%), friends (15.3%), and medical staff (1.4%) may also affect the psychology of COVID-19 patients.

Depression (58.2%), fear (55.2%), loneliness (48.5%), nervousness (51.8%), sadness (12.7%), worry (52.8%), loss of interest in daily activities (46.2%), sleep issues (60.9%), and memory loss/inattention (18.3%) also affect the psychological pain of COVID-19 patients.

Issues related to the patients' appearance/bodily form (5.0%), bathing/dressing (7.6%), breathing (57.2%), micturition changes (2.6%), physical constraints (7.8%), diarrhea (1.4%), eating issues (6.3%), tiredness (28.6%), edema (1.0%), fever (72.8%), dizzy (8.2%), independence (9.1%), oral pain (5.4%), nausea (2.0%), dry mucous membranes (5.4%) or congestion (60.1%), pain (19.2%), sexual dysfunction (2.0%), dry/itchy skin (0.8%), numbness of hands/feet (3.4%), and limited physical activity (3.0%) also affect their psychological pain levels.

Spiritual/religious concerns (2.4%) also affected psychological pain in the patients sampled in this study.

## Discussion

### Correlation Analysis of Influencing Factors, Psychological Pain and PTG in COVID-19 Patients

Through the data analysis in Table 2, it can be concluded that the higher the score, the more posttraumatic growth. This is consistent with previous work by Lili et al. (2010).

Through the data analysis in Table 3, it can be concluded that the sampled COVID-19 patients' psychological pain was negatively correlated with their total PTG score. There was a significant negative correlation between the total score of PTG and psychological pain. This is consistent with the study of Dandan (2019).

Through the data analysis in Table 4, it can be concluded that males appear to suffer significantly more psychological pain than females, which is consistent with previous observations made by Xu et al. (2020). Middle-aged patients may need to bear more family-related burdens than other age groups which more severely impact their quality of life after contracting the disease. That is, older patients appear to suffer a higher the degree of psychological pain than younger patients. Again, Xu et al. (2020) reached a similar conclusion. When COVID-19 first began to spread, China did not have a corresponding national relief policy. This phenomenon has had a huge negative impact on low-income patients. Because they are under financial pressure to visit a doctor, and because of the suspension of the epidemic, they cannot complete their work, which makes them lose their financial income. Taking the average monthly income as the measurement standard, patients with monthly income of 2,000–3,000 CNY (309.6-464.4 USD) have the greatest psychological pain, which is consistent with previous work by Xueqin et al. (2019). Long-term medical isolation prevents patients from direct contact with their relatives and friends, which is known to cause significant psychological pain. The psychological pain grew more intense as the patient's isolation time increased. This is consistent with the results of Xueying (2014). That is, more serious disease experiences produced a higher level of psychological pain. This is consistent with the results of Sujuan et al. (2018). Patients in the full or partial knowledge group had a lower degree of psychological pain than those who were ignorant of the disease, which is consistent with the results of Min et al. (2018). That is, more-educated patients and those with greater knowledge of COVID-19 show less intense psychological pain. This is consistent with previous observations by Min et al. (2018). A more scientific, rational, and informed understanding of COVID-19 may reduce the panic and pressure caused by “the unknown,” thus decreasing the patient's level of psychological pain. The psychological pain score in the very poor health group was significantly higher than in other groups. This is consistent with the results of Sujuan et al. (2018). Patients with a history of mental illness had a higher degree of psychological pain, which was consistent with the results of Hao et al. (2019). Mental illness can affect the cognitive function of patients to the extent that they are more inclined toward higher psychological pain compared to patients with no history of mental illness. It is possible that marriage simply does not affect the psychological condition of patients. Past medical history may not influence present psychological pain due simply to the length of time from the past to the present.

Through the data analysis in Table 5, it can be concluded that patients struggling with care for the old adultsly and children, financial problems, and work/school problems showed significant psychological pain, which is consistent with the results of Xueqin et al. (2019). Illness can disrupt one's normal family life and cut off sources of income, placing significant pressure on the patient in regards to sustaining his or her family. Resulting changes in the quality of life of the patients' family can increase psychological pain. Patients with depression, fear, loneliness, tension, sadness, worry, loss of interest in daily life, and sleep issues showed significant psychological pain, which is consistent with the results of Wenlan et al. (2019). Discomforting emotional experiences, physical discomfort, and resulting changes to one's work and home life can cause physical and mental injury. For patients with COVID-19, negative emotions undoubtedly aggravate any pre-existing psychological pain. Patients with respiratory, fatigue, fever, and dryness/congestion showed significant psychological pain, which is consistent with previous observations by Jie et al. (2019). The negative effects on the body brought on by COVID-19 may drive down the patient's quality of life and intensify his or her psychological pain. Patients with less economic resources may struggle with medical expenses, which increases psychological pain is (Xueqin et al., 2019). Patients unable to perform housework or other family-related tasks, in addition to fear that the economic burden of their illness will impact their family, also show a high degree of psychological pain (Xueqin et al., 2019). Patients also may suffer changes to their abilities to work and study due to the disease—or may fear losing their job or falling behind in school—which creates significant psychological pain (Roger et al., 2019). COVID-19 can create a particular sense of pessimism due to the current lack of long-term effective control and treatment (Juan and Qinglian, 2008). Patients with communication problems show higher levels of psychological pain. COVID-19 treatment necessitates isolation, which creates and/or exacerbates communication problems. A lack of contact or real/perceived lack of support from friends, family, and medical staff intensifies patients' psychological pain (Zhanjiang, 2020). Patients with negative emotions (e.g., sadness) suffer more over the course of the disease than patients whose mental states are more stable or optimistic (Wenlan et al., 2019). Negative emotions undoubtedly aggravate psychological pain in COVID-19 patients. Sleep issues commonly comorbid with mental illness can result in excessive fatigue, a perceived loss of control, and changes in attention and memory which increase the patient's level of psychological pain (Qingsong, 2013; Liqian et al., 2020). Patients who lose interest in life also experience greater psychological suffering while experiencing COVID-19 (Zhuoyan, 2020). All manner of negative bodily manifestations may increase the patient's level of psychological pain (Jie et al., 2019; Shuzhe et al., 2020). Respiratory problems are also negative manifestations of COVID-19 specifically. Patients with respiratory problems showed higher levels of psychological pain than others. Breathing is, of course, a necessary metabolic process of the human body. Issues with breathing can create significant psychological pain (Zhuoyan, 2020). The proportion of patients with sexual dysfunction is very low in our sample, which may be due to the physical and psychological pain they experienced during their illness having prevented them from focusing on their sex lives in general. Further, Hao et al. (2019) showed that poor cognitive function intensifies psychological pain; patients with previous mental problems showed lower cognitive function and thus more intense psychological pain in this study as well.

**Table 5:** Influencing factor analysis.

**Influence factor**		**Total**	**Composition ratio (%)**	**DT ≥ 5**	**DT < 5**	***X*^**2**^**
Practical problem	Child and old adults care	92	18.6	72	20	5.783^*^
	Housing	47	9.4	22	25	0.377
	Insurance/financial	182	36.7	125	57	0.148^**^
	Transportation	10	2.1	2	8	0.128
	Work/school	305	61.5	63	242	4.466^*^
	Surrounding environment	253	51.0	137	116	0.400
Communication problems	Children/Old adults	52	10.4	23	29	0.041
	Partner	98	19.8	44	54	2.369
	Friends	76	15.3	31	45	0.551
	Medical staff	7	1.4	1	6	0.682
Emotional problems	Depression	289	58.2	201	88	9.942^***^
	Fear	274	55.2	196	78	0.948^**^
	Loneliness	241	48.5	177	64	6.564^***^
	Nervousness	257	51.8	174	83	5.710^**^
	Sadness	63	12.7	52	11	8.540^*^
	Worry	262	52.8	191	71	5.548^**^
	Loss of interest in daily activities	229	46.2	142	87	5.262^*^
	Sleep	302	60.9	218	84	11.335^**^
	Memory loss/inattention	91	18.3	55	36	2.396
Physical problems	Appearance/form	25	5.0	19	6	1.742
	Bathing/dressing	38	7.6	27	11	1.806
	Breathing	284	57.2	187	97	1.136^**^
	Micturition changes	13	2.6	4	9	0.762
	Constipation	39	7.8	27	12	1.128
	Diarrhea	7	1.4	1	6	0.627
	Eating	31	6.3	18	13	4.161
	Tiredness	142	28.6	94	48	4.041^**^
	Edema	5	1.0	1	4	0.639
	Fever	361	72.8	244	117	5.107^**^
	Dizzy	41	8.2	22	19	2.705
	Indigestion	45	9.1	24	21	0.126
	Oral pain	27	5.4	12	15	0.112
	Nausea	10	2.0	2	8	2.705
	Dry membranes/congestion	298	60.1	185	113	4.621^*^
	Pain	95	19.2	62	33	1.534
	Sexual	10	2.0	4	6	0.041
	Dry/itchy skin	4	0.8	0	4	0.192
	Numbness of hands/feet	17	3.4	4	13	2.940
	Limited physical activity	15	3.0	6	9	0.772
Spiritual/religious concerns	Spiritual/religious concerns	12	2.4	5	7	0.019

* *p < 0.05*,

** *p < 0.01*,

*** *p < 0.001*.

### COVID-19 Related Psychological Pain and Correlation With Post-traumatic Growth

COVID-19 has an incubation period of 1–14 days. There is a lack of etiological evidence regarding the disease due to its novelty; treatments have been developed based on other similar diseases. Suspected or confirmed cases are subjected to long-term observation, which can have a substantial psychological impact on patients (Lu et al., 2020). The psychological pain score of COVID-19 patients in this study was 6.89 (4.32 + 2.57), which falls within the moderate pain level range. It is important to evaluate the psychological pain of patients with COVID-19 after treatment to formulate scientific and effective psychological interventions.

The COVID-19 patients' PTG score was (53.58 ± 8.45), which suggests that the PTG level of COVID-19 patients is relatively low. Pearson's correlation analysis showed that the total PTG score, life perception, interpersonal relationships, personal strength, mental changes, and new possibility factors were negatively correlated with the psychological pain of patients with COVID-19 (*p* < 0.05). At the significance level of *p* < 0.05, the correlation between the two groups was negative.

This result is consistent with previously published results. Qing et al. (2018), for example, measured psychological pain and PTG levels in patients with cervical cancer after chemotherapy; parallel correlation analysis showed a negative correlation between them. Conversely, Lechner et al. (2003) found that the degree of threat perceived by patients over the course of their disease is positively correlated with post-traumatic outcomes. The relationship between psychological pain and PTG may not be a simple linear relationship, but an inverted U-shaped relationship. When psychological pain reaches a certain level, it shows a positive correlation with PTG which then becomes negative once the pain exceeds a critical point. COVID-19 may create stronger negative emotions because it is a novel disease with which neither patients nor medical professionals have previous experience. Liyan and Yuhan (2019) found that cancer remission has a positive psychological reaction to PTG which improves the physical and mental health, and thus the overall quality of life, for survivors.

### Factors Influencing Psychological Pain in COVID-19 Patients

Based on COVID-19 patients' baseline data, disease information, and psychological pain factors, chi square were conducted to determine the effects of illness severity, surrounding environment, family problems, life perception, interpersonal relationships, personal strength, mental changes, new possibilities, and post-traumatic outcomes. The disease itself was the main factor influencing the psychological pain of COVID-19 patients (*p* < 0.05, *p* < 0.01).

Factors increasing the severity of psychological pain, from high to low, were dangerous cases, severe cases, common cases, and mild cases. In effect, the degree of psychological pain was highest in patients with dangerous cases (*p* < 0.05). COVID-19 has clinical manifestations of varying severity which are treated differently; patients experiencing severe symptoms may be placed on ventilators to prevent hypoxia, which is psychologically distressing (Huang et al., 2020). The initial stages of COVID-19 are mainly characterized physically by fatigue and weakness and mentally by a distressing lack of knowledge about the disease and the measures that will be necessary to treat it. Patients with severe cases, compared to those with mild or dangerous cases, may feel more supported by medical professionals as they receive regular treatment over the course of the disease; this perception of stable and effective support can mitigate the psychological pain they experience. The drugs given to patients with mild to moderate symptoms do not cause as much pain or distress as the ventilators and other interventions necessary for severe manifestations of the disease.

COVID-19 indeed creates psychological pain in patients (*p* < 0.05). Once any member of a family becomes a COVID-19 patient, their role in the family unit changes dramatically. The necessity to self-isolate during treatment can be extremely discomforting. COVID-19 patients are confronted with mortality daily as they navigate the course of the disease, which comes at a significant psychological cost and can create intense sadness.

COVID-19 is also highly infectious, even among asymptomatic persons, which is psychologically burdensome. Infection routes include personal contact, aerosolized respiratory droplets, and feces. Individuals must self-isolate if they suspect that they have been exposed to the virus through any of these routes. When an individual family member receives a diagnosis, they must accept that it is likely they have exposed the rest of their family to the virus; the potential threat to loved ones and the self-isolation necessary to protect others lead to high levels of psychological pain.

COVID-19 patients reported psychological pain related to several independent risk factors in this study (*p* < 0.05). The results reveal a negative correlation between COVID-19 patients' psychological pain and PTG. COVID-19 patients must be isolated during treatment and receive medication, surgery, breathing machines, and any other necessary interventions while unable to be in physical proximity to loved ones, which seriously affects their quality of life and elevates their psychological pain. COVID-19 patients' mental health levels should be checked even after they are discharged from the hospital to guide them as they experience any PTG and as they readjust to their normal lives.

### Research Limitations

The sample size of this study is relatively small due to the conditions of the disease and its spread across China. The foreign scale was also not localized. Although the work was revised based on China's national conditions, there are still limitations.

## Conclusion

This study suggests that there was a significant negative correlation between the total score of PTG and psychological pain. There was a significant negative correlation between psychological pain and posttraumatic growth. There are significant differences in the degree of psychological pain among different gender, age, education level, income, occupation and marriage. Practical problems, communication problems, emotional problems, physical problems, and spiritual/religious concerns have significant effects on the degree of psychological pain.

Suspected and confirmed cases of COVID-19 continue to affect the lives of countless people around the world. The virus has caused severe psychological trauma to those patients who eventually overcome the disease after long-term isolation treatment. Even a moderate degree of psychological pain can seriously affect the physical and mental health of patients. By fully understanding the influencing factors of patients' psychological pain, health staff can analyze the psychological status of patients to help design the necessary aftercare.

## Data Availability Statement

The raw data supporting the conclusions of this article will be made available by the authors, without undue reservation.

## Ethics Statement

Ethical review and approval was not required for the study on human participants in accordance with the local legislation and institutional requirements. Written informed consent to participate in this study was provided by the participants.

## Author Contributions

NZ contributed to theory construction, writing the paper, and revising the paper. SY assisted in revising the paper. All authors contributed to the article and approved the submitted version.

## Conflict of Interest

The authors declare that the research was conducted in the absence of any commercial or financial relationships that could be construed as a potential conflict of interest.
